# Supplementary material for: Antibodies inhibit transmission and aggregation of C9orf72 poly‐GA dipeptide repeat proteins
Source: EMBO Mol Med. 2017 Mar 28;9(5):687–702. doi: 10.15252/emmm.201607054 (PMC5412769; doi:10.15252/emmm.201607054)
Supplement: Supplementary file 1 — Expanded View Figures PDF [file EMMM-9-687-s001.pdf]

Expanded View Figures

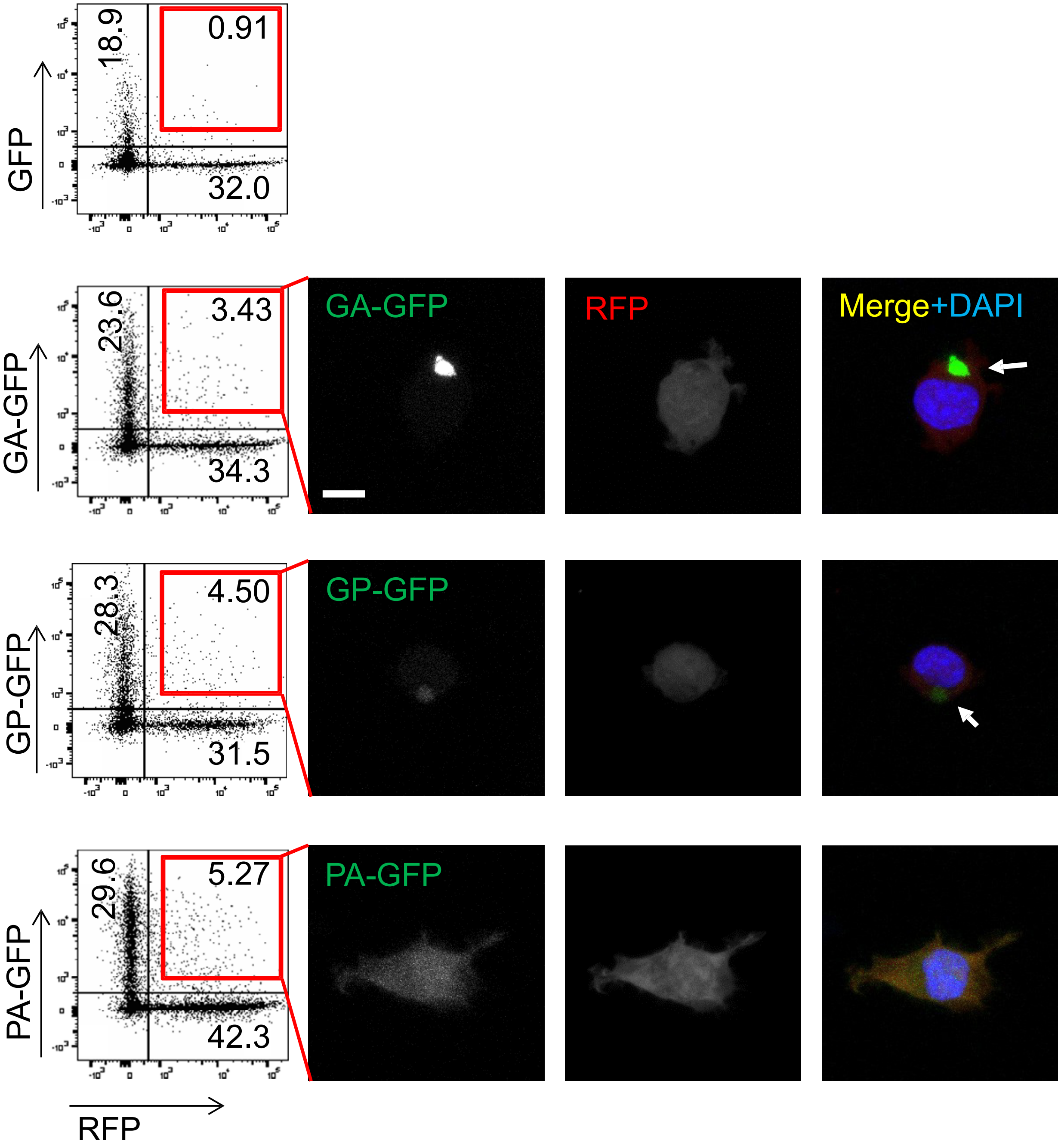

Figure EV1. Transmission of hydrophobic DPR proteins in a co-culture assay.
HEK293 cells were transfected with RFP, GFP, or DPR-GFP for 24 h and mixed in the indicated combination for additional 24 h as in Fig 2 before cell sorting by flow cytometry. Gating was performed on RFP-expressing cells vs. mixture of all green fluorescent cells. The fraction of double-positive cells is indicated in percent. Double-positive cells were sorted and plated on poly-D-lysine-coated coverslips and imaged 17 h later. Images show uptake of DPR-GFP into RFP-positive cells. Arrows indicate co-localization of GA_{175}-RFP aggregates with GA_{175}-GFP and GP_{47}-GFP. Scale bar 10 μm.

© 2017 The Authors

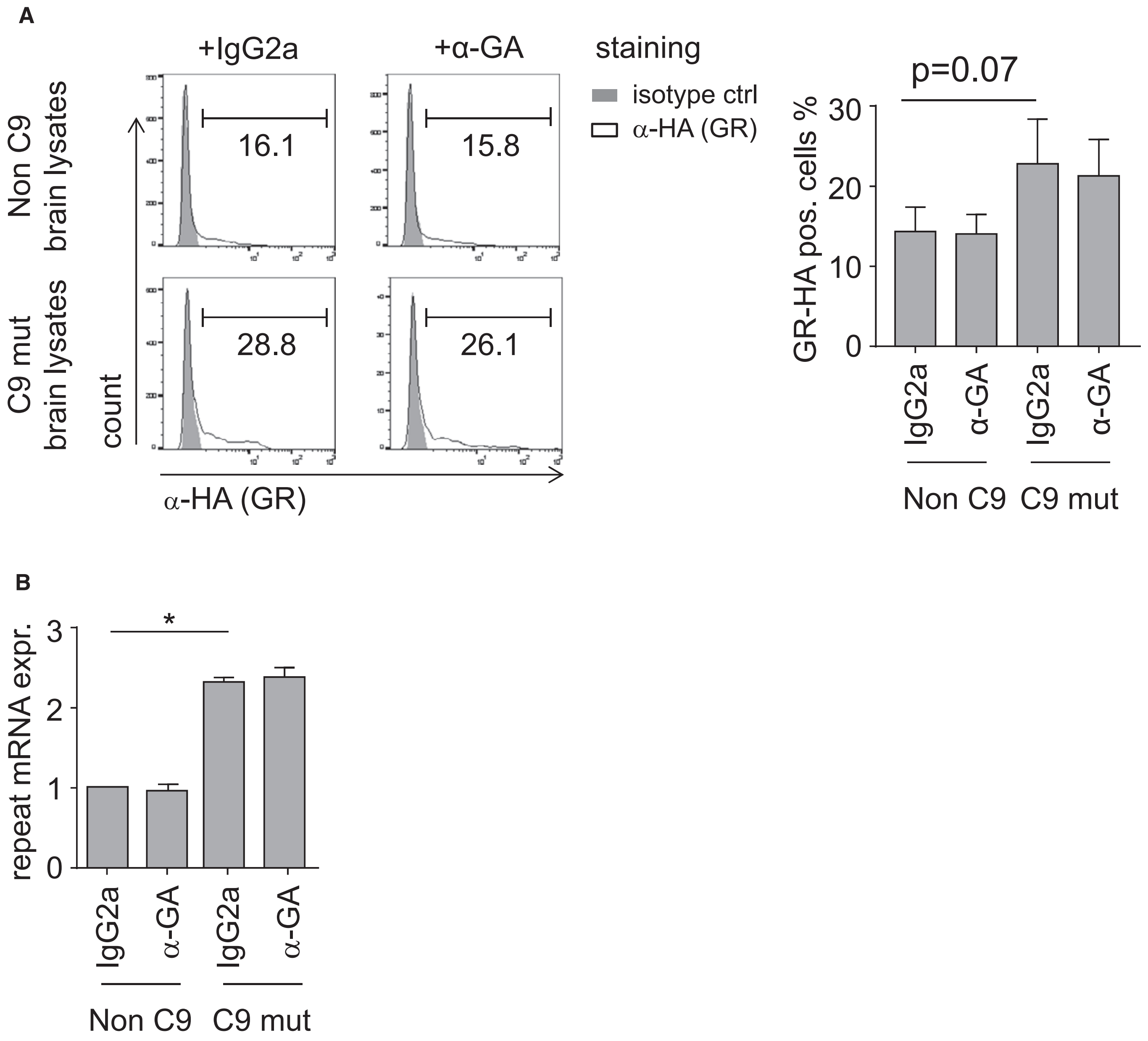

Figure EV2. Anti-GA antibodies do not reduce expression of poly-GR and repeat RNA.
HEK293 cells transfected with $(G4C2)_{80}$ were treated with cerebellar extracts pre-incubated with anti-GA or isotype control.

A The fraction of RAN translation-derived GR_{80}-HA was quantified by flow cytometry. Data indicated the means ± SD of n = 3 patients and controls in independent experiments. Statistics were performed by one-way ANOVA with Dunnett's multiple comparisons test.

B Quantitative RT–PCR shows repeat RNA transcripts upon treatment with cerebellar extracts pre-incubated with anti-GA or isotype control. Data are shown as mean ± SD from n = 3 patients and controls in independent experiments. Statistics were performed by one-way ANOVA with Dunnett's multiple comparisons test; non-C9 + IgG2a vs. C9 mut + IgG2a P = 0.0168; *P < 0.05.

 © 2017 The Authors
